# Myometrial thickness overlying cesarean scar pregnancy is significantly associated with isthmocele formation in the third month of the postoperative period

**DOI:** 10.4274/tjod.galenos.2021.65288

**Published:** 2021-03-12

**Authors:** Resul Karakuş, Sultan Seren Karakuş, Burak Güler, Gökhan Ünver, Enis Özkaya

**Affiliations:** 1 University of Health Sciences Turkey, Zeynep Kamil Women and Children’s Diseases Training and Research Hospital, Clinic of Obstetrics and Gynecology, İstanbul, Turkey

**Keywords:** Isthmocele, scar pregnancy, cesarean, myometrial thickness

## Abstract

**Objective:**

To determine some associated factors for isthmocele formation 3 months after the treatment of cesarean scar pregnancy (CSP).

**Materials and Methods:**

This is a prospective consecutive case series of CSP managed by fertility preservation modalities at a single tertiary care center from May 2016 to March 2019 (n=95). Patients with a diagnosis of CSP were identified and followed prospectively to collect data on the patients’ demographics; detailed medical, surgical, and social history; symptoms; imaging and laboratory parameters at the time of CSP diagnosis and during treatment; treatment modalities, myometrial thickness; and outcomes in terms of isthmocele formation.

**Results:**

Mean myometrial thickness overlying scar pregnancy was significantly lower in the group with isthmocele formation, and the mean gestational age of scar pregnancy was also significantly lower in the group with isthmocele formation following treatment of scar pregnancy (p<0.05). Multivariate regression analysis was conducted to determine associations between certain variables and isthmocele development, which revealed that the gestational age of scar pregnancy and myometrial thickness were significantly associated with isthmocele formation.

**Conclusion:**

Myometrial thickness and gestational age of scar pregnancy were significantly associated with isthmocele formation 3 months after treatment.

**PRECIS:** Myometrial thickness and gestational age of scar pregnancy were significantly associated with isthmocele formation 3 months later after treatment.

## Introduction

Pregnancy in a previous cesarean scar occurs in approximately 1 in 2.000 pregnancies and constitutes 6% of ectopic pregnancies among women who had a previous cesarean delivery^(1,2,3)^. The incidence does not appear to be related to the number of cesarean births, similar pregnancies have been reported in the literature, including pregnancies implanted in previous myomectomy scars^(4)^. Pregnancy is located on the scar and is surrounded by myometrium and connective tissue. The mechanism of implantation to this site is believed to be the migration of the embryo through a wedge defect in the lower uterine segment or through a microscopic fistula in the scar^(5,6)^. In symptomatic patients, the clinical appearance ranges from painful or painless vaginal bleeding to uterine rupture and hypovolemic shock^(7,8)^. The diagnosis is made sonographically (transvaginal and transabdominal) by visualizing an enlarged hysterotomy scar with a buried mass that can extend beyond the anterior contour of the uterus^(9,10)^. The optimal treatment for a cesarean scar pregnancy (CSP) is unclear and therapy should be tailored to the patients’ clinical presentation. Treatment options include wedge resection of the ectopic pregnancy via laparotomy or laparoscopy, or possible hysterectomy, dilatation, and curettage or methotrexate therapy. In subsequent pregnancies, recurrent scar implantation may occur^(11)^. There are reports of successful term pregnancy after a CSP^(11)^. Isthmocele is a myometrial defect that looks like a pouch on the anterior wall of the uterine isthmus above the previous cesarean scar^(12)^. Isthmocele formations were shown to be a risk factor for cesarean scar ectopic pregnancy^(13)^.

This study aimed to determine some associated factors for isthmocele formation at the third month following treatment of scar pregnancy.

## Materials and Methods

This is a prospective consecutive case series of CSP managed by fertility preservation modalities at a single tertiary care center (University of Health Sciences Turkey, Zeynep Kamil Women and Children’s Health Training and Research Hospital) from May 2016 to March 2019 (n=95). The study protocol was approved by the institutional review board (University of Health Sciences Turkey, Zeynep Kamil Women and Children’s Health Training and Research Hospital Ethics Committee -2017/05) and written informed consent was obtained from each participant. The patients with a diagnosis of CSP were identified and followed prospectively to collect data on patients’ demographics: detailed medical, surgical, and social history; symptoms; imaging and laboratory parameters at the time of CSP diagnosis and during treatment; treatment modalities, myometrial thickness and outcomes in terms of the presence of isthmocele formation at third postoperative month and successful pregnancy following treatment. All diagnoses were made based on the patient’s history of prior cesarean delivery, positive pregnancy test, presence of a gestational sac in the area of the scar, and otherwise empty uterine cavity on transvaginal ultrasonogram. Either a medical or surgical method was used as a treatment modality determined based on demographic and clinical characteristics (ultrasonography findings, beta-human chorionic gonadotropin (hCG) level, fetal cardiac activity ±) of each individual. Medical management consisted of a single dose methotrexate regimen (50 mg/m^2^ body surface area); the second systemic methotrexate dose was given to the patients who declined surgical management when their first dose had failed. Dilatation and curettages were performed under general anesthesia with ultrasonography guidance, following ensuring removal of gestational material, a 20-F Foley catheter was placed in the uterine cavity to control heavy bleeding if it occurred. Postoperatively, the patient was monitored in the intensive care unit. As alternative management of CSP, hysteroscopy was performed after cervical dilatation, and hysteroscopic scar pregnancy removal was performed with bipolar energy. In patients who required an extracavitary approach, a laparoscopy was performed following a uterine manipulator insertion. The bladder and adhesions were dissected. CSP was removed using bipolar energy. The scar edges were expanded with scissors without any energy modalities. The incision was closed using continuous double-layer sutures.

All participants underwent myometrial thickness measurement of the scar area (Mindray DC-7) by the same sonographer. Myometrial thickness was defined as the minimum thickness overlying the amniotic cavity at the level of the uterine scar. In the postoperative third month, participants were reevaluated for isthmocele and risk of scar pregnancy recurrence.

The diagnosis of isthmocele formation at postoperative third month was established using transvaginal ultrasound as previously described^(14)^, performed using a 5-MHz transvaginal transducer (Mindray DC-7) 3 to 6 days after the last menstruation by the same sonographer. An anechoic triangle defect in the myometrium with the base communicating to the uterine cavity, or a deformity on the anterior isthmus was considered to be isthmocele^(15)^.

### Statistical Analysis

Data analysis was performed using the SPSS version 15.0 package (Chicago, IL). Student’s t-test and the Mann-Whitney U test were used to compare continuous variables. The chi-square and Fisher’s exact tests were used for categorical variables. Receiver operating characteristics analysis was used to determine predictive values. Multivariate regression analysis was used to show adjusted associations. P-values <0.05 were accepted to be statistically significant.

## Results

### Comparison of Outcome Variables

There were 56 (58.9%) cases with isthmocele formation detected in the third postoperative month. There were 23 (24.2%) healthy pregnancies among the study population after postoperative follow-up. The rate of isthmocele formation in the third postoperative months was significantly higher in the group without pregnancy (65% vs 39.1%, p=0.03).

### Comparison of Variables with and Without Isthmocele

**Figure 1:**
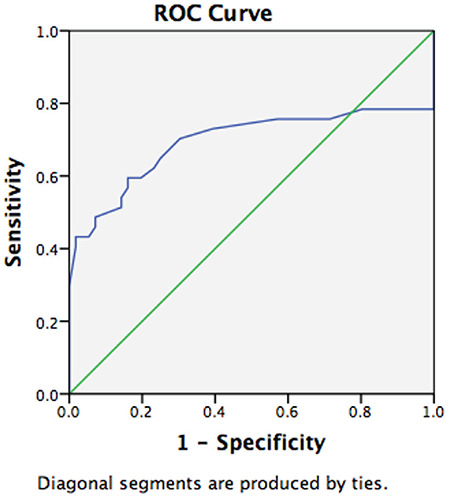
ROC analysis of myometrial thickness to predict postoperative isthmocele ROC: Receiver operating characteristics

Groups with and without isthmocele formation following treatment of scar pregnancy were compared in terms of age, gravidity, parity, number of previous cesarean deliveries, and body mass index, and analysis of the data revealed no differences between the groups in terms of these variables (Table 1, p>0.05). A comparison of groups with and without isthmocele formation following treatment of scar pregnancy in terms of myometrial thickness and gestational sac diameter resulted in significant differences between the groups. The mean myometrial thickness was significantly lower in the group with isthmocele following treatment of scar pregnancy, and the mean gestational age of scar pregnancy was also significantly lower in the group with isthmocele formation following treatment of scar pregnancy (Table 2, p<0.05). Myometrial thickness was a significant predictor for isthmocele formation following treatment of scar pregnancy [area under the curve (AUC)=0.693, p=0.002]. The optimal cut-off value was 4.1 mm with 70% sensitivity and 70% specificity (Figure 1).

### Treatment Modalities

**Figure 2:**
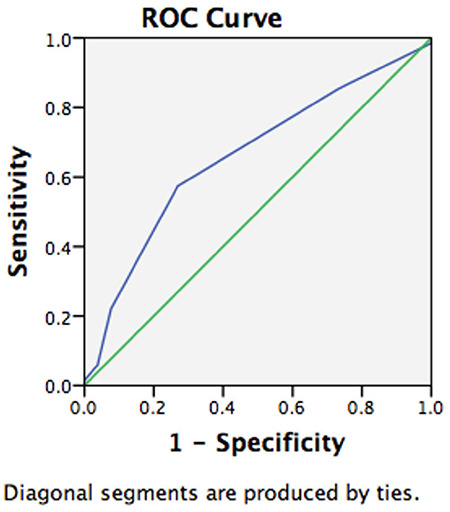
ROC analysis of gravidity to predict the need for further intervention following failed uterine curettage ROC: Receiver operating characteristics

A comparison of groups with and without further treatment following failed curettage revealed significant differences between the groups in terms of gravidity and age (Table 3, p<0.05). Among 26 patients who required further intervention secondary to failed curettage, the management modalities were as follows: laparoscopic scar pregnancy removal and scar closure (n=14), methotrexate (n=8), and hysteroscopic scar pregnancy removal and uterine cavity revision (n=4). Gravidity was a significant predictor for the failure of treatment with uterine curettage alone (AUC=0.660, p=0.02). The optimal cut-off value was 3.5 with 57% sensitivity and 73% specificity (Figure 2). The rate of isthmocele at the third postoperative month was 65.4% in the group that underwent further intervention following failed uterine curettage, whereas it was 56.5% in patients who were treated with uterine curettage (p=0.434).

### Multivariate Regression Analysis to Show Adjusted Associations

Multivariate regression analysis was conducted to determine associations between certain variables and isthmocele formation, and the analysis revealed that the gestational age of scar pregnancy [odds ratio (OR): 0.4, 95% confidence interval (CI): (0.2-0.89), p=0.005] and myometrial thickness [OR: 0.6, 95% CI: (0.5-0.9), p=0.006] were significantly associated with isthmocele formation.

### Associations Between Variables and Symptoms at Admission

**Figure 3:**
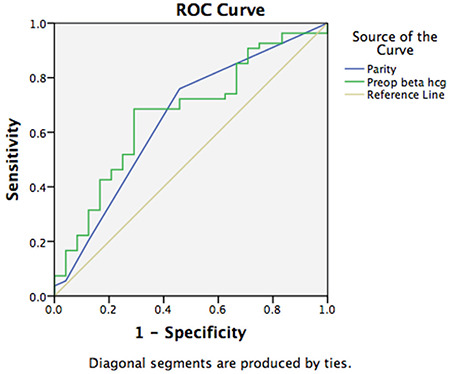
ROC analysis of parity and beta-hCG to predict vaginal bleeding as the symptom of the first admission ROC: Receiver operating characteristics

A comparison of the groups with and without vaginal bleeding before intervention showed a significant difference between the two groups in terms of age, gravidity, parity, and preoperative beta-hCG levels (p<0.05). Parity (AUC=0.650, p=0.04, optimal cut-off value=1.5 with 73.4% sensitivity, 57% specificity) and preoperative beta-hCG levels (AUC=0.667, p=0.02, optimal cut-off value=10.700 with 69% sensitivity, 71% specificity) were significant predictors for vaginal bleeding before intervention (Figure 3).

## Discussion

In this case series, we aimed to determine some associated factors for isthmocele formation in the third month following treatment of scar pregnancy. Our data analysis revealed that myometrial thickness overlying CSP and gestational age of scar pregnancy were significantly associated with isthmocele formation 3 months after treatment. The incidence and diagnosis of CSP are rapidly increasing, mainly due to higher cesarean rates and increased use of ultrasound in early pregnancy. Early diagnosis and treatment of CSP are important to increase the success rate of treatment and prevent complications^(16)^. Vaginal bleeding after amenorrhea was the most common but non-specific symptom. Some patients with CSP may experience low abdominal pain and vaginal bleeding simultaneously^(17)^. There were 28 (29.5%) cases of vaginal bleeding at first admission in our study population; a comparison of groups with and without vaginal bleeding before intervention showed a significant difference between the two groups in terms of age, gravidity, parity, and preoperative beta-hCG levels. Parity and preoperative beta-hCG levels were significant predictors for vaginal bleeding before the intervention.

Various methods have been proposed for the management of CSP without consensus on the optimal treatment method. Options include local and/or systemic medical therapy with methotrexate, uterine artery embolization, and surgical procedures such as D&C, laparoscopic or hysteroscopic gestational mass resection and hysterectomy^(18,19)^. Surgical treatment has been described with a success rate of 83%, but with a complication rate of 18% compared with 7% for medical treatment^(18)^. In a recent national cohort study in the United Kingdom, surgical treatment was identified with a 96% success rate but a 36% complication rate^(20)^.

Given that there is no consensus on the optimal treatment modality of CSP, several treatment modalities have been compared in the literature in terms of success and complication rates. The most frequently assessed modalities were expectant management, D&C with the guidance of ultrasound, direct injection of potassium chloride into the embryonic sac with the guidance of ultrasound, local or systemic injection of methotrexate^(21)^, uterine artery embolization, hysteroscopy, and laparotomy or laparoscopic excision^(22,23)^. However, none of these treatments was found to be entirely satisfactory. Success and complication rates of three different modalities including transvaginal clearance, endoscopic surgery, uterine artery embolism were compared in a study by Fei et al.^(17)^, and the authors concluded that early detection of CSP and conservative treatment greatly improved the prognosis of patients and suggested transvaginal pregnancy tissue clearance may be the preferred option for a fertility protection approach. In their study, blood loss was the lowest with transvaginal pregnancy tissue clearance among the three groups. For this procedure, there is no need to enter the pelvic cavity so pelvic adhesions have no adverse effect during the surgical course. On the other hand, it was shown that resection of the old scar with a new uterine closure may reduce the recurrence of scar dehiscence^(24)^. Endoscopic surgery includes hysteroscopy or laparoscopy or a combination of these two modalities. In the majority of cases in our series, D&C was successful in the management of scar pregnancy cases. Surgical resection of the scar may be considered to be associated with a lower risk of scar pregnancy recurrence or isthmocele formation; however, our analysis failed to show any difference among different management modalities in terms of isthmocele formation.

Uterine artery embolization was also suggested as a treatment option for CSP, which could block the blood flow of uterine arteries, decrease vascularization, and induce trophoblastic degeneration. In previous studies, uterine artery embolization resulted in satisfactory results when combined with local methotrexate^(25)^. Because uterine artery embolization may interfere with the ovarian reserve, it cannot be a suitable choice for women who want to preserve fertility^(26)^.

In a study published in 2016, the efficacy of ultrasound-guided suction curettage for the management of pregnancies implanted into the lower uterine segment cesarean section scar was assessed in 232 women with cesarean section scar pregnancy. The authors showed that ultrasound-guided transcervical surgical evacuation was an effective method for the treatment of first-trimester CSP. There were no cases of uterine perforation in their series, but the proportion of women diagnosed with retained products of conception on postprocedure ultrasound examination was higher when compared with women who underwent surgical evacuation of failed intrauterine pregnancies^(27)^.

In another study, the risk of bleeding was shown to be increased with advancing pregnancies, but the authors stated that vascularity of the pregnancy on Doppler examination was the most significant predictor of excessive blood loss, obstruction of the cervical canal using a Foley catheter helps to control bleeding following the evacuation of CSPs^(28)^.

In the majority of the cases in our series, D&C was successful in the management of scar pregnancy cases. Gravidity was a significant predictor for the failure of treatment, the success rate of D&C increased with higher gravidity. A Foley catheter was used in only a few cases to control bleeding.

It is now well known that one of the most common gynecologic sequelae of c-section is a uterine scar with deficient healing, known as an isthmocele or c-section defect^(29)^. The poor contractility of the myometrium around the isthmocele caused by the presence of fibrotic tissue can produce a blood drainage deficiency with the accumulation of blood during the menstrual cycle at the level of the scar and subsequent spotting^(29)^, normally during the first week of the cycle.

Previous data showed that isthmocele contributes to the development of cesarean scar ectopic pregnancy^(13)^ and isthmoplasty was suggested to be an option to prevent the occurrence of a scar ectopic pregnancy, thereby preventing massive blood loss and allowing the conservation of the uterus to maintain fertility, health, and quality of life^(30)^. The success of hysteroscopic surgery on isthmocele associated with CSP was reported by some authors. It was shown that hysteroscopic surgery was effective in increasing the residual myometrial thickness and reducing the size of isthmocele^(31)^. As the women in our series desired to preserve their fertility, isthmocele formation was critical for the possible future pregnancy. In our case series, myometrial thickness overlying scar pregnancy and gestational age of scar pregnancy were found to be significantly associated with isthmocele formation. On the other hand, the type of scar pregnancy management modality was not associated with the isthmocele formation at the postoperative third month.

This was a prospective cohort study of scar pregnancies, the relatively large sample size based on a single-center data and the evaluation of various outcomes of interest and independent variables were the major advantages of this study. The major disadvantage of this study was the lack of data regarding the rate of isthmocele before scar pregnancy.

## Conclusion

Myometrial thickness and gestational age of scar pregnancy were significantly associated with isthmocele formation 3 months after treatment. Myometrial thickness measurement overlying scar pregnancy may be used to select candidates for further intervention following treatment of scar pregnancy.

## Figures and Tables

**Table 1:**
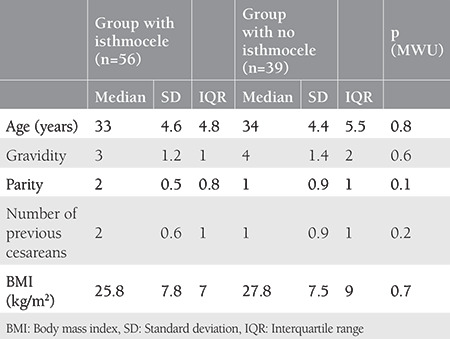
Groups with and without isthmocele formation following treatment of scar pregnancy compared in terms of age, gravidity, parity, number of previous cesarean deliveries, and BMI

**Table 2:**
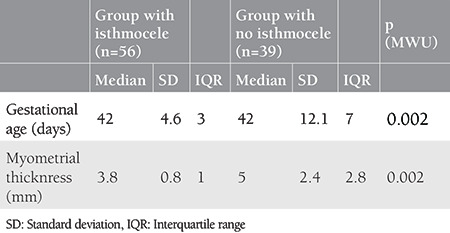
Comparison of groups with and without isthmocele formation following treatment of scar pregnancy in terms of myometrial thickness and gestational sac diameter

**Table 3:**
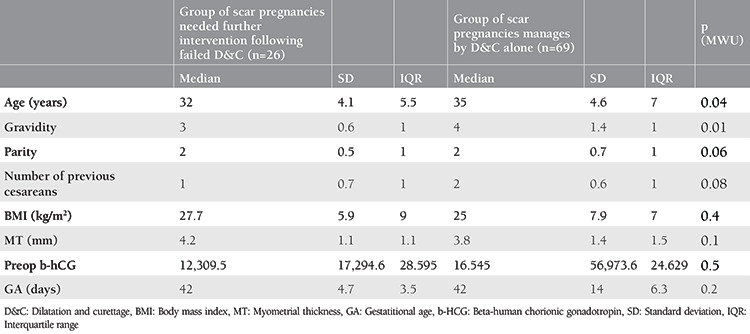
Comparison of groups with and without further treatment following failed curettage
